# Depression and Anxiety in Patients with Gastroesophageal Reflux Disorder With and Without Chest Pain

**DOI:** 10.7759/cureus.6103

**Published:** 2019-11-08

**Authors:** Saleh Mohammad, Bashir Chandio, Aftab A Soomro, Salma Lakho, Zamanat Ali, Zulifqar Ali Soomro, Faizan Shaukat

**Affiliations:** 1 Gastroenterology, Ghulam Muhammad Mahar Medical College and Hospital, Sukkur, PAK; 2 Internal Medicine, Ghulam Muhammad Mahar Medical Hospital, Sukkur, PAK; 3 Pathology, Ghulam Muhammad Mahar Medical Hospital, Sukkur, PAK; 4 Internal Medicine, Ghulam Muhammad Mahar Medical College and Hospital, Sukkur, PAK; 5 Orthopaedics, Ghulam Muhammad Mahar Medical Hospital, Sukkur, PAK; 6 Internal Medicine, Jinnah Postgraduate Medical Centre, Karachi, PAK

**Keywords:** gerd, gastro-esophageal reflux disease, depression, anxiety

## Abstract

Introduction

Gastroesophageal reflux disease (GERD) influences patients’ general health, daily and social functioning, and physical and emotional activities. It strongly affects the health-related quality of life with frequent interruptions during sleep, work, and social activities. GERD is defined as a condition that develops when the reflux of stomach contents causes troublesome symptoms and/or complications. GERD symptoms are a major concern for many patients, as they cause a disturbance in physical, social and emotional health. In this study, we determine the prevalence of anxiety and depression in patients with GERD with and without chest pain.

Methods

In this cross-sectional study, a total of 258 consecutive patients with a diagnosis of GERD were included in this study. Of 258 participants, 112 had concerns about chest pain. Clinical presentations and comorbid disorders were evaluated by a previously validated gastroesophageal reflux symptom questionnaire. Depressive and anxious symptoms were assessed using a Hospital Anxiety/Depression Scale.

Results

A total of 107 (41.4%) participants had depression, 89 (34.4%) participants had anxiety, and 70 (27.13%) had both depression and anxiety. Depression and anxiety were significantly higher in patients with GERD and chest pain.

Conclusion

Anxiety and depression were significantly higher in patients with GERD, particularly those who also reported concerns of chest pain. Measures should be taken to reduce the stress and anxiety of GERD patients to cope with their daily life activities and improve their quality of life.

## Introduction

Gastroesophageal reflux disease (GERD) has a strong impact on the health-associated quality of life (QOL), affecting daily social activities and physical and emotional well-being of patients. GERD also interferes with healthy sleep and work. GERD is a digestive disorder that affects the lower esophageal sphincter, causing irritating symptoms like heartburn or acid indigestion [1]. The symptoms of GERD appear to be a great load for patients with regard to physical, emotional, and social health [2]. GERD is also one of the main problems of the upper gastrointestinal (GI) tract; 10% to 20% of the population reports symptoms of GERD every week. From 2005 to 2010, symptoms-based GERD was prevalent among 5.2% to 8.5% of the population in Eastern Asia. In Iran, it was prevalent among 6.3% to 18.3% of the population, as Iran is the only country in South East Asia where the majority of studies on the prevalence of GERD have been performed. In Pakistan, the studies based in hospitals have revealed that GERD is prevalent in 22.2% and 24.0% of the population in two different local studies [3]. GERD and its complications, including Barrett’s esophagus and adenocarcinoma of the esophagus, are increasing in prevalence worldwide [4]. The symptoms of GERD are irritating and can even expand from the usual main symptoms of regurgitation and pyrosis [1].

The association between functional GERD and psychological health has been identified in many studies [5, 6]. A close connection may exist between the GI tract and the brain. For instance, the occurrence of emotional distress and tension can have affect GI function leading to GI diseases. In the same way, GI stress can affect mental and emotional well-being. The severity of a functional GI disorder can be affected by psychological elements, altering the perception of pain through activity on the gut-brain axis, which also applies to GERD.

Moreover, psychological factors increase the difficulty in treating functional GI disorder, leading to poor outcomes [5]. However, there was no significant association between the severity of symptoms of GERD and pathophysiological irregularities as identified by 24-hour pH and esophageal manometry. This supports the concept that GERD symptoms are greatly influenced by psychological factors [7]. To the best of our knowledge, few published global reports establish a relationship between psychological factors, including depression and anxiety, and GERD symptoms to date and the outcomes of those studies are not consistent [8].

There should be a clear picture of the relationship between psychological factors and GERD so that appropriate treatment can be determined and administered in GERD patients, as psychological factors can escalate the symptoms of GERD along with worsening the results of the therapy and interfering with QOL. This study, therefore, focuses on identifying the prevalence of depression and anxiety in GERD patients.

## Materials and methods

In this cross-sectional study, 258 consecutive patients with a diagnosis of GERD were included from January 1, 2019, to July 30, 2019. Of 258 participants, 112 reported concerns of chest pain. All patients provided informed verbal consent and were invited to complete a standardized questionnaire. This investigation was approved by the Local Ethical Committee for Ghulam Muhammad Mahar Medical College. 

Gastroesophageal clinical presentation was evaluated on a gastroesophageal reflux symptom questionnaire. In this questionnaire, esophageal and extraesophageal symptoms related to GERD were assessed. A five-item Likert scale was used to grade the frequency and severity of symptoms. GERD was diagnosed on clinical symptoms, such as pyrosis (i.e., heartburn) and regurgitation, according to the Montreal standard. A seven-item, that was validated by Yang XJ in his study, GERD questionnaire was used for the diagnosis of GERD, and a cut-off of 12 was recommended for a specificity of 84% and a sensitivity of 82% [9].

Depressive and anxious symptoms were assessed using a Hospital Anxiety/Depression Scale (HADS), which has robust psychometric properties and is brief and easy to administer. The HADS consists of 14 items divided into two 21-point subscales for anxiety and depression, and a score of ≥ 8 was considered abnormal for either anxiety or depression [9].

The data were entered and analyzed using IBM SPSS Statistics for Windows, Version 21.0. (Armonk, NY: IBM Corp.). Frequencies and percentages were calculated for descriptive variables including age, gender, body mass index, and smoking status. Independent t-tests were applied to compare mean, and chi-square was applied to compare categorical data. A p-value of 0.05 was considered significant.

## Results

In this cross-sectional study, 258 consecutive patients with GERD were included. Of them, 112 (43.4%) reported concerns of chest pain. GERD patients with chest pain were significantly but not substantially older than GERD patients without chest pain. The GERD scores were comparable in both groups (Table 1).

**Table 1:** Demographics of participants BMI - body mass index; GERD - gastrointestinal esophageal reflux disease; SD - standard deviation

Demographics	GERD without chest pain (n=146)	GERD with chest pain (n=112)	p-value
Age, years (mean ± SD)	44 ± 13	51 ± 11	0.0001
Gender (male/female)	78/68	60/52	---
BMI, kg/m^2^ (mean ± SD)	23.43 ± 5.22	24.12 ± 3.81	0.23
Smoking, n (%)	36 (24.6)	31 (27.67)	---
GERD score	15.34 ± 3.49	15.68 ± 3.78	0.455

A total of 107 (41.4%) participants had depression, 89 (34.4%) participants had anxiety, and 70 (27.13%) had both depression and anxiety. Depression and anxiety were significantly higher in patients with GERD and chest pain (Table 2).

**Table 2:** Hospital Anxiety and Depression Score GERD - gastrointestinal esophageal reflux disease; HADS - Hospital Anxiety and Depression Score

HADS score	GERD without chest pain (n=146)	GERD with chest pain (n=112)	p-value
HADS depression	52 (35.6%)	55 (49.1%)	0.02
HADS anxiety	35 (23.9%)	54 (48.2%)	0.000
HADS depression and anxiety	30 (24.7%)	40 (35.2%)	0.006

## Discussion

We found high levels of depression and anxiety in GERD patients with and without chest pain. Anxiety and depression levels were significantly higher in patients with chest pain than in those without chest pain. This may be due to the patient’s perception that chest pain is a sign of serious ailment, which may contribute to higher levels of psychological burden which includes anxiety and depression [9]. This result was also repeated by Yang et al., who stated that physical symptoms, particularly chest pain, can have detrimental effects on mental status. They reported anxiety and depression to be more common in patients with chest pain as opposed to those without it [10]. In our study, patients with GERD without chest pain and GERD with chest pain had a depression prevalence of 35.6% and 49.1%, respectively. Similarly, the anxiety reported for patients with GERD and chest pain and GERD without chest pain was 24.7% and 35.17%, respectively. This result was consistent with the findings of Zhang et al. [9].

The full correlation between anxiety and depression with GERD is not fully understood. However, a close relationship between the brain and GI tract has been established. Stress and emotions can alter GI function and can even cause GI symptoms and disease. Based on this premise and the available data, diseases of GI symptoms may also affect emotional status. Psychological factors may affect the severity of a GI disorder by affecting the perception of pain through the gut-brain axis. This mechanism is also applicable to patients with GERD [5]. It is important to address the psychological factors that accompany GERD, as the treatment of GERD in the presence of psychological effects can become hindered [5]. Kimura et al. stated that patients who did not respond to proton pump inhibitors were more likely to show higher levels of anxiety and depression [11].

While our study was first to highlight the prevalence of depression and anxiety among patients with GERD in Pakistan, it has a few limitations. First, because it was a single-center study, the result cannot be generalized to a broader population. It was a cross-sectional study, so we cannot determine the exact direction of correlation between GERD and psychological symptoms (i.e. if GERD caused psychological symptoms or vice versa). The response to treatment was not noted.

## Conclusions

This cross-sectional study suggests a high prevalence of anxiety and depression among patients with GERD. The adverse psychological effects were more significant in patients who reported concerns of chest pain. GERD imposes negative effects on QOL, which can both exacerbate and be exacerbated by depression and anxiety. A multidisciplinary approach is needed to manage GERD and its associated psychological symptoms to reduce the impact of the disease on QOL and mental health. If patients are non-responsive to conventional treatment, the use of anti-anxiety and anti-depression medications should be considered. This particular subject needs more attention, and large-scale prospective trials are needed to explore this potential relationship further.
