# Correction: Depression and Anxiety in Patients with Gastroesophageal Reflux Disorder With and Without Chest Pain

**DOI:** 10.7759/cureus.c25

**Published:** 2019-12-10

**Authors:** Saleh Mohammad, Bashir Chandio, Aftab A Soomro, Salma Lakho, Zamanat Ali, Zulifqar Ali Soomro, Faizan Shaukat

**Affiliations:** 1 Gastroenterology, Ghulam Muhammad Mahar Medical College and Hospital, Sukkur, PAK; 2 Internal Medicine, Ghulam Muhammad Mahar Medical Hospital, Sukkur, PAK; 3 Pathology, Ghulam Muhammad Mahar Medical Hospital, Sukkur, PAK; 4 Internal Medicine, Ghulam Muhammad Mahar Medical College and Hospital, Sukkur, PAK; 5 Orthopaedics, Ghulam Muhammad Mahar Medical Hospital, Sukkur, PAK; 6 Internal Medicine, Jinnah Postgraduate Medical Centre, Karachi, PAK

The third column of Table 2 incorrectly repeated the second column header 'GERD without chest pain' instead of the correct 'GERD with chest pain'.

The Table header has been revised to correct this error and is now correctly labeled 'GERD with chest pain'.

